# Ankyrin Repeat Domain 1 Protein: A Functionally Pleiotropic Protein with Cardiac Biomarker Potential

**DOI:** 10.3390/ijms18071362

**Published:** 2017-06-26

**Authors:** Samantha S. M. Ling, Yei-Tsung Chen, Juan Wang, Arthur M. Richards, Oi Wah Liew

**Affiliations:** 1 Cardiovascular Research Institute, National University Health System, Singapore 119228, Singapore; mdcslsm@nus.edu.sg (S.S.M.L.); mdccyt@nus.edu.sg (Y.-T.C.); deliawj@gmail.com (J.W.); 2 Department of Medicine, Yong Loo Lin School of Medicine, National University of Singapore, 14 Medical Drive, Singapore 117599, Singapore; 3 Christchurch Heart Institute, University of Otago, Christchurch 8014, New Zealand

**Keywords:** ankyrin repeat domain 1, cardiac mechano-sensing, cardiomyopathy, heart failure, biomarker

## Abstract

The ankyrin repeat domain 1 (ANKRD1) protein is a cardiac-specific stress-response protein that is part of the muscle ankyrin repeat protein family. ANKRD1 is functionally pleiotropic, playing pivotal roles in transcriptional regulation, sarcomere assembly and mechano-sensing in the heart. Importantly, cardiac ANKRD1 has been shown to be highly induced in various cardiomyopathies and in heart failure, although it is still unclear what impact this may have on the pathophysiology of heart failure. This review aims at highlighting the known properties, functions and regulation of ANKRD1, with focus on the underlying mechanisms that may be involved. The current views on the actions of ANKRD1 in cardiovascular disease and its utility as a candidate cardiac biomarker with diagnostic and/or prognostic potential are also discussed. More studies of ANKRD1 are warranted to obtain deeper functional insights into this molecule to allow assessment of its potential clinical applications as a diagnostic or prognostic marker and/or as a possible therapeutic target.

## 1. Introduction

Ankyrin repeat domain 1 (ANKRD1), also known as cardiac ankyrin repeat protein (CARP), belongs to the conserved muscle ankyrin repeat protein (MARP) family. The MARP family has two other members, namely ankyrin repeat domain protein 2 (ANKRD2/ARPP) and diabetes-related ankyrin repeat protein (ANKRD23/DARP) [1]. MARPs have dual nuclear-cytoplasmic localization in the cell [1] but their tissue distributions differ: ANKRD1 is predominantly expressed in the heart [2]; ANKRD2 mainly in skeletal muscle [3]; and DARP is equally present in both [4].

The expression of ANKRD1 is high in the early embryonic heart but is down-regulated to relatively lower levels in the adult heart [5]. This differential expression profile of ANKRD1 suggests a role in cardiogenesis where it has been implicated as a negative transcriptional regulator of cardiac gene expression [2]. ANKRD1 is also expressed in fetal skeletal muscle but is barely detectable in adult skeletal muscle [3]. Upon activation by hypertrophic stimuli and during heart failure, ANKRD1 expression in cardiomyocytes is highly and rapidly up-regulated, suggesting its importance in pathological cardiac remodeling [6,7]. In this paper, we review the properties, functions and regulation of ANKRD1 and highlight current gaps in knowledge. The potential of ANKRD1 as a cardiac biomarker is also further discussed.

## 2. Gene and Protein Characteristics

ANKRD1 is encoded by the *ANKRD1* gene located on chromosome 10 in humans and is highly conserved among mammals [8]. The cDNA of *ANKRD1* is 1901 base pairs long and encodes a protein product consisting of 319 amino acids (aa) with a theoretical molecular weight of 36,252 Da [9]. Alternative splicing of *ANKRD1* by intron retention events has also been observed in human cardiomyocytes [10]. In particular, two isoforms of *ANKRD1* (*ANKRD1-i8* and *ANKRD1-i7,8*) were detected but their functional significance remains unclear.

**Figure 1:**
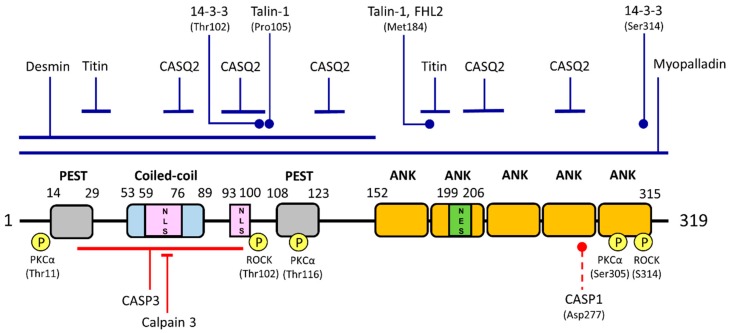
Schematic representation of human ankyrin repeat domain 1 (ANKRD1) structure, binding partners and phosphorylation and cleavage sites. The ANKRD1 protein has 319 amino acids consisting of a coiled-coil domain, two PEST-like regions, an ankyrin repeat domain consisting of five ankyrin repeats (ANK), two putative nuclear localization signals (NLSs) and one potential nuclear export signal (NES). ANKRD1 has two binding sites for titin and five binding sites for calsequestrin (CASQ2) which are indicated by blue solid lines. Regions or residues involved in interaction with myopalladin, desmin, talin-1, four-and-a-half LIM domains 2 (FHL2) and 14-3-3 proteins are also indicated accordingly. Experimentally verified phosphorylation sites (yellow-circled P) and the protein kinases (PKCα, protein kinase C; ROCK, Rho kinase) responsible are indicated. Regions harboring cleavage sites by caspase-3 (CASP3) and calpain 3 are indicated by solid red lines. Predicted caspase-1 (CASP1) cleavage site is marked by a dashed line. Diagram is not drawn to scale.

The ANKRD1 protein consists of several distinctive features: (1) coiled-coil domain; (2) tandem ankyrin repeats; (3) nuclear localization signature motifs; (4) PEST protein degradation sequences; and (5) putative post-translational modification sites (Figure 1). A single coiled-coil domain is located near the N-terminal region of ANKRD1 and is predicted to be between residues 53 and 89 using the software COILS v2.2 (http://www.ch.embnet.org/software/COILS_form.html) [11]. Coiled-coil motifs typically consist of a supercoil of two to five α-helices that are wrapped around each other and are structurally able to mediate the oligomerization of proteins [12]. In addition, the coiled-coil domain of ANKRD1 has been demonstrated to contribute to its self-dimerization although the interaction is relatively weak [13]. More recent work showed that in addition to homo-dimerization, ANKRD1 also forms hetero-dimers with ANKRD2/ARPP and ANKRD23/DARP via their coiled-coil domains [14].

**Figure 2:**
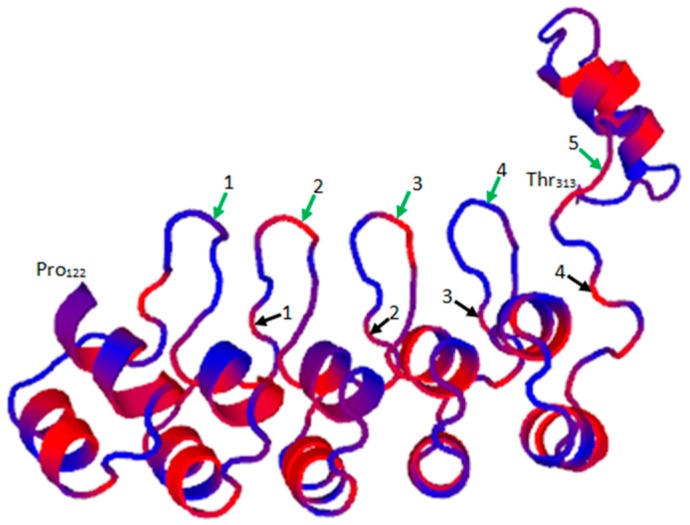
Predicted Swiss Model of ANKRD1. The 3D structure of ANKRD1 from Pro_122_ to Thr_313_ is shown (blue) with hydrophobic residues indicated in red. Ankyrin repeat units 1 (Tyr_152–_Phe_181_), 2 (Leu_185_–Ala_214_), 3 (Leu_218_–Ala_247_), 4 (Glu_251_–Ile_280_) and 5 (Ala_284_–Arg_315_) are indicated by green and black arrows showing the start and end of each unit, respectively. The end of ankyrin repeat unit 5 terminating at Arg_315_ is not shown.

ANKRD1 has five predicted tandem ankyrin repeats present at the C-terminus region of the protein (UniProtKB/Swiss-Prot Entry Q15327; http://www.uniprot.org/uniprot/Q15327). Homology modeling of the ANKRD1 sequence against ANK-N5C-281 (PDB entry 4qfv.2.B; https://swissmodel.expasy.org/templates/4qfv.1) as template using Swiss Model (https://swissmodel.expasy.org/) revealed typical ankyrin repeat units comprising a β-turn followed by two anti-parallel α-helices and an overall right-handed solenoidal structure with a slight curvature (Figure 2). The helix structures of each repeat unit are packed against those of the adjacent repeat and are stabilized by hydrophobic interactions at the interface between repeats as well as hydrogen bond networks that connect the β-hairpin/loop regions. The ankyrin repeat units comprise 30–32-residue sequence motifs implicated in mediating protein-protein interactions. Ankyrin repeats are abundantly present in a variety of proteins with diverse functions including transcriptional regulation, cell-cycle regulation, ion transport, cytoskeleton integrity, endocytosis and signal transduction, among many others [15,16].

ANKRD1 is predicted to possess two nuclear localization signals (NLS; specific peptide sequences that contain a cluster of basic aa such as lysines or arginines) [17], indicating that it could be actively transported into the nuclei of cells. ANKRD1 has been observed in the nuclei of rat cardiomyocytes [18]. It is worth noting that ANKRD1 has a predicted nuclear export signal located within the second ankyrin repeat domain of ANKRD1 as predicted using the software NetNES v1.1 (http://www.cbs.dtu.dk/services/NetNES/) [19]. However, there is no report on the active transport of ANKRD1 out of the nucleus to date.

ANKRD1 possesses two potential PEST sequences at its N-terminus region [17]. PEST motifs are regions rich in proline (P), glutamic acid (E), serine (S) and threonine (T) that serve as proteolytic signals [20]. Proteins with these degradation sequences typically have short intracellular half-lives due to their rapid degradation rates.

Bioinformatic analyses have predicted multiple putative phosphorylation sites in ANKRD1 that are phosphorylated by protein kinase C, cyclic adenosine monophosphate-dependent protein kinase and casein kinase II [2]. Experimentally, using in vitro kinase assays, ANKRD1 was indeed found to be phosphorylated at several sites (Thr11, Thr116 and Ser305) by protein kinase C [14]. ANKRD1 is not phosphorylated by protein kinase A. Phosphorylation of ANKRD1 by Rho kinase has also been documented at Thr102 and/or Ser314 [21]. Other post-translational modifications that have been predicted to be present in ANKRD1 include *N*-glycosylation, *N*-myristoylation and amidation [2]. However, none of these post-translational modifications have been experimentally verified. Furthermore, in silico analysis using NetNGlyc v1.0 (http://www.cbs.dtu.dk/services/NetNGlyc/) and SignalP v4.0 (http://www.cbs.dtu.dk/services/SignalP/) on the Expasy bioinformatics resource database predicts that ANKRD1 does not contain a signal peptide directing its translocation into the endoplasmic reticulum [22,23]. Hence, ANKRD1 may not be exposed to the glycosylation machinery in cells. Future studies on this aspect of ANKRD1 may yield more information on its functions and/or regulation.

## 3. Ankyrin Repeat Domain 1 Localization, Interacting Partners and Related Functions

### 3.1. Localization

ANKRD1 displays dual subcellular localization: in the nucleus of the myocyte as well as in the cytoplasm within the sarcomere and displays multiple functions correlating to its localization [1]. In the nucleus, ANKRD1 acts as a regulator of gene expression of target genes while in the cytoplasm, it interacts with sarcomeric proteins to maintain sarcomere organization [1,24]. Cytoplasmic ANKRD1 has also been reported to be shuttled into the nucleus upon induction by stimuli such as mechanical stretch [1]. Thus, it has been proposed that sarcomeric ANKRD1 functions as part of a stretch-sensing unit capable of relaying biomechanical stress signals to the regulation of gene expression [25].

ANKRD1 has been reported to bind to a large variety of proteins which is not surprising given its pleiotropic functions [26]. In a recent report, Glutathione *S*-transferase (GST)-tagged ANKRD1 was found to pull down 493 candidate binding proteins, including ANKRD1 itself [21]. However, it is worth noting that some of these binding partners may not be interacting directly with ANKRD1 itself but are instead, part of a complex involving ANKRD1. In this section, we center on the interacting partners of ANKRD1 which have been experimentally verified and focus on their significance in relation to the functions of ANKRD1 with emphasis on implications on cardiac function in health and disease (summarized in Table 1).

### 3.2. Interaction with Transcription Factors: Regulation of Gene Expression

#### 3.2.1. Y-Box Binding Protein 1 (YB-1)

One of the first interacting partners of ANKRD1 that was discovered is Y-box binding protein 1 (YB-1) [18], a transcription factor that has been implicated in the regulation of gene expression [34]. Using the yeast two-hybrid approach where YB-1 was used as bait, it was found that ANKRD1 formed a complex with the former [18]. This interaction was further confirmed using co-immunoprecipitation and GST pulldown assays in the same study. One of the functions of YB-1 includes being a positive regulator of ventricular specific myosin light chain-2 (*MLC2v*) gene, a protein which is important in cardiogenesis. Interestingly, ANKRD1 acts as a negative transcriptional co-factor of YB-1 where it has been proposed to sequester YB-1 to antagonize and regulate its functions.

#### 3.2.2. Nuclear Factor κ-Light-Chain-Enhancer of Activated B Cells (NF-κB)

Another transcription factor which ANKRD1 binds to and represses is nuclear factor κ-light-chain-enhancer of activated B cells (NF-κB) [27]. In the study by Liu et al., they demonstrated using co-immunoprecipitation that ANKRD1 binds to the p50 subunit of NF-κB and vice versa in differentiating C2C12 myoblasts. This ANKRD1-NF-κB interaction leads to repression of NF-κB transcriptional activity induced by tumor necrosis factor-α (TNF-α), thus suggesting an anti-inflammatory role of ANKRD1. In addition, NF-κB p65 DNA binding activity was also reported to be reduced when ANKRD1 was overexpressed in C2 mouse myotubes [17]. Hence, it was postulated that ANKRD1 may either bind to NF-κB, resulting in the latter’s cytoplasmic sequestration, or act on upstream signaling pathways that regulate NF-κB nuclear activity.

#### 3.2.3. Nucleolin

ANKRD1 interacts with nucleolin, a transcriptional regulator of target genes controlled by the AP-1 binding element on their promoter regions [28]. Association of ANKRD1 with nucleolin hinders the binding of positive transcriptional activators to the AP-1 site, resulting in the inhibition of *matrix metalloproteinase 13* (*MMP13*) gene transcription. Although MMPs play a major regulatory role in the wound repair process, their overexpression however often leads to pathological outcomes due to uncontrolled degradation of the extracellular matrix [35,36]. Thus, it was hypothesized that ANKRD1 limits the expression of MMP13 to appropriate physiological levels to enable successful tissue repair [28]. Indeed, ANKRD1 is strongly elevated after wounding which suggests its role in the healing process [37]. Furthermore, it was recently shown that wound closure was significantly delayed in *Ankrd1* knockout mice [38], lending further support to the role of ANKRD1 in tissue repair.

#### 3.2.4. Tumor Suppressor p53

Not all functions of ANKRD1 on transcription factors are repressive in nature. Using protein array methodology, p53 was identified as another interacting partner of ANKRD1 and this interaction was shown to occur in vitro and in vivo [29]. In this case, ANKRD1 acts as a positive transcriptional co-activator of p53, moderately up-regulating its activity and the subsequent downstream expressions of p21 (a cell cycle inhibitor), murine double minute 2 (Mdm2) and ANKRD2 in cultured skeletal muscle cells, suggesting its regulatory roles during development and stress response.

The above transcription factors are only a handful out of those that have been identified to be interacting partners of ANKRD1. In the protein array performed by Kojic et al., at least 16 other transcription factors implicated in a wide range of functions were also found to interact with ANKRD1 [29]. Some examples include Jun proto-oncogene (c-Jun), histone deacetylase 1 (HDAC1) and mothers against decapentaplegic homolog 3 (Smad3). Further studies confirming their interactions as well as their functional significance would be an interesting area to examine and would enhance our understanding of the multiple functions of ANKRD1.

### 3.3. Interaction with Structural Components of the Sarcomere: Maintenance of Sarcomere Integrity and Mechano-Sensing Functions

#### 3.3.1. Myopalladin and Titin

ANKRD1 binds to two myofibrillar proteins within the I-band of striated muscle sarcomeres, namely myopalladin and titin [1,24]. Myopalladin is a cytoskeletal protein whose central proline-rich IS3 region binds to skeletal muscle nebulin’s and cardiac muscle nebulette’s Src homology 3 (SH3) domain [24]. In the study by Bang et al., it was found that myopalladin also binds to ANKRD1 and sarcomeric α-actinin at its N- and C-terminal domains respectively. Their data further suggested that the ANKRD1-myopalladin interaction is essential for maintaining sarcomeric structure as overexpression of myopalladin’s N-terminal ANKRD1-binding domain led to a severe disruption of sarcomeric components in live cardiac myocytes. Interestingly, it was noted that only full length ANKRD1 interacted with myopalladin as the binding was ablated when 5′ and 3′ deletions of ANKRD1 were introduced. The role of ANKRD1 in maintaining sarcomere structural integrity was further supported by a separate study where it was shown that siRNA-knockdown of *Ankrd1* in adult rat ventricular myocytes resulted in marked sarcomere disarray compared to control cells [39].

Titin (also known as connectin) is a giant filamentous polypeptide that functions to maintain structural integrity in sarcomeres as well as provide passive stiffness to striated muscle by acting as a molecular spring [40]. A single titin molecule binds to the Z and M line of the sarcomere, spanning half its length. ANKRD1 has been demonstrated to bind to the elastic N2A region of titin via two titin-binding sites, one at its N-terminal and the other within its ankyrin repeat region [1]. Importantly, this ANKRD1-myopalladin-titin complex has been proposed to function as a mechano-sensing machinery in cardiac myocytes, whereby ANKRD1 trans-locates to the nucleus upon stretch to regulate gene transcription [1]. The exact mechanism as to how this occurs has not been fully elucidated to-date and would be an exciting area to further investigate.

#### 3.3.2. Desmin

Using yeast two-hybrid screening, desmin (also a coiled-coil protein) was demonstrated to interact with the N-terminal region of ANKRD1 [13]. Desmin is a muscle-specific, major intermediate filament protein found in cardiac, skeletal and endothelial cells. It is found mainly in the Z-disk of striated muscles and maintains the muscle cyto-architecture structure by forming a scaffold around the Z-disk and connecting it with the subsarcolemmal cytoskeleton, nuclei and other organelles [41]. The significance of the ANKRD1-desmin interaction, however, is as yet unknown.

#### 3.3.3. Talin-1

ANKRD1 was also found to bind to talin-1, a cytoskeletal protein found in costamere structures in cardiac and skeletal muscle cells [42]. Talin-1 mediates cell-cell adhesion by linking integrins to the actin cytoskeleton and is also a putative mechano-sensory unit [8,43]. The functional significance of ANKRD1 binding to talin-1 has not been fully elucidated. However, it has been suggested to play a role in cellular mechanical stretch-based gene regulation as disruption of ANKRD1-talin 1 interaction resulted in an enhanced down-regulation of p53 and an up-regulation of myogenin expression [8].

### 3.4. Interaction with Signaling Molecules

#### 3.4.1. Four-And-A-Half LIM Domains 2 (FHL2)

Binding of ANKRD1 and four-and-a-half LIM domains 2 (FHL2) was demonstrated using yeast two-hybrid assay [8]. FHL2 is a transcription co-factor that is highly expressed in the heart although it has also been found in other tissue types [44]. Each LIM domain of FHL2 contains two zinc finger motifs which have various binding specificities to DNA, RNA, proteins and lipids [45]. However, it is presently unclear whether FHL2 binds to ANKRD1 via its zinc finger domains. FHL2 can be found in the titin-N2B and β-integrin complexes and has also been proposed to function as a molecular transmitter that integrates different signaling pathways through the regulation of transcription [44,46]. Like other interacting partners of ANKRD1, the functional relevance of ANKRD1-FHL2 interaction remains presently unclear and should be further investigated.

#### 3.4.2. Calsequestrin 2 (CASQ2)

Using immunoprecipitation and pulldown assays, it was found that ANKRD1 binds to the cardiac calsequestrin (CASQ2) [32]. CASQ2 is a calcium-binding protein found in the sarcoplasmic reticulum of cardiac muscle cells. It functions to bind and store sufficient Ca^2+^ in the sarcoplasmic reticulum to allow for repetitive contractions and is crucial for heartbeat and respiration [47]. Five CASQ2 binding sequences on ANKRD1 and three ANKRD1 binding regions on CASQ2 have been mapped [32]. However, it is still unclear how this coupling can take place in vivo since ANKRD1 has not been reported to be found in the sarcoplasmic reticulum. Nonetheless, it has been proposed that CASQ2 can be transiently found in the cytoplasm and that ANKRD1-CASQ2 interaction at the I band (where ANKRD1 is anchored) could lower Ca^2+^ concentration to effect various signaling pathways [32].

#### 3.4.3. 14-3-3 Proteins

In the recent study by Yura et al., it was reported that ANKRD1 also binds to three species of 14-3-3 proteins (ε, γ and ζ) [21]. 14-3-3 proteins belong to a conserved family of regulatory molecules that bind a wide range of signaling proteins to modulate their activities, act as steric regulators or function as a scaffold protein to mediate protein-protein interactions [48]. Association of ANKRD1 with these 14-3-3 proteins occurred in a phosphorylation-dependent manner whereby the phosphorylated form of ANKRD1 resulted in a 10-fold or higher increased intensity (as measured by mass spectrometry) compared to the non-phosphorylated form [21]. Binding of 14-3-3 to ANKRD1 was also found to contribute to the cytoplasmic retention of ANKRD1. Interestingly, the authors speculate that phosphorylation of ANKRD1 by Rho kinase at Thr102 promotes binding of 14-3-3 protein to ANKRD1 at this site, thereby masking the effect of the nearby NLS on ANKRD1 and hence influencing its subcellular localization.

#### 3.4.4. Protein Kinase C Alpha (PKCα)

In a recent study, it was demonstrated by GST-pulldown and co-immunoprecipitation assays that ANKRD1 forms a signalosome complex with ANKRD2, phospholipase C β1 and protein kinase C alpha (PKCα) at the intercalated disc [33]. Interestingly, this assembly only occurred in dilated cardiomyopathy and failing hearts and was not observed in wild type hearts. These results showed that the relocation of ANKRD1 from the sarcomere leads to persistent sequestration of PKCα at the intercalated disc, resulting in chronic PKCα signaling in failing hearts leading eventually to heart failure. It is worth noting that in the same study, knockout of *Ankrd1* was able to rescue the phenotype of muscle LIM protein (MLP)-deficient mice. This finding suggests a potential of ANKRD1 as a therapeutic target for cardiomyopathy which should be further studied.

### 3.5. Effects of Ankrd1 Knockout in Mice

Given the multiple functions of ANKRD1 in the heart including regulation of gene expression and mechano-sensing properties as described, it is surprising that mice with single knockout of *Ankrd1*, *Ankrd2* or *Ankrd23* and even triple knockout mice for the three *Marp* genes were found to be viable and display normal cardiac function under basal conditions as well as in response to pressure overload [49]. It is unclear whether this result is species-dependent and/or limited to the mode of stimulation used (that is, transverse aortic constriction). This finding is unexpected considering the many studies indicating the involvement of ANKRD1 in sarcomere assembly, mechano-stretch sensing, nuclear gene regulation, cardiogenesis and cardiac disease. It is noteworthy that total knockout of myoglobin, a key oxygen transporter and scavenger of bioactive nitric oxide in the heart and muscle [50], in mice did not result in any abnormal phenotype and these animals remain viable and fertile [51]. These transgenic animals exhibit normal cardiac function and are well adapted even to severe hypoxic stress [52]. Compensatory mechanisms have also been reported in these myoglobin-deficient mice to preserve cardiac function [53,54,55]. It is tempting to speculate that the lack of functional and structural consequences in MARP-deficient mice may be the result of “buffering” mechanisms afforded by other proteins. Although the role of ANKRD1 in normal cardiac function is presently controversial, it remains clear upregulation of ANKRD1 expression in response to cardiac insults is apparently associated with important pathological effects.

## 4. Pathological Cardiac Functions of ANKRD1

### 4.1. Hypertrophic Stress Responses

The expression of cardiac ANKRD1 is rapidly increased in response to various hypertrophic stimuli including pressure overload and mechanical stress [1,6]. Accordingly, ANKRD1 has been observed to be highly induced in cardiac hypertrophy [6]. It has been suggested that induction of ANKRD1 is an adaptive and protective response against various stresses as ANKRD1-over-expressing transgenic mice displayed markedly decreased cardiac hypertrophy in response to pressure overload or continuous isoproterenol infusion compared to wild type mice [56]. The attenuation of cardiac hypertrophy by ANKRD1 was also found to involve the inhibition of ERK and TGF-β/Smads signaling pathways by ANKRD1 in the same report. However, it is interesting to note that in another study by Chen et al. [57], increased expression of ANKRD1 using recombinant adenoviral vectors carrying *Ankrd1* in mice with transverse aortic constriction exacerbated pathological cardiac remodeling through activation of the calcineurin/nuclear factor of activated T-cells (NFAT) pathway, a signaling pathway known to have an important role in cardiac hypertrophy [58]. In support of the pathological role of ANKRD1, it was observed that *Ankrd1* null mice did not display cardiac hypertrophy in response to phenylephrine while wild type mice showed significant hypertrophy due to ANKRD1-induced hypertrophic gene expression, indicating that ANKRD1 could be involved in accelerating the progression of hypertrophy [25]. Possible reasons for the seemingly contradictory results from these studies include the differing levels of over-expression of ANKRD1 in transgenic vs. Ad-*Ankrd1* infected mice and whether this overexpression is persistent or transient. In addition, whether ANKRD1 overexpression or pressure overload occurs first may also contribute to different outcomes. Therefore, the role of ANKRD1 with regards to hypertrophy is still controversial. Whether ANKRD1 functions to enhance or inhibit hypertrophy should be further studied with these factors in mind.

### 4.2. Cardiac Fibrosis

Cardiac fibrosis, a common pathological manifestation in myocardial infarction and heart failure, involves adverse fibroblast accumulation and excessive deposition of extracellular matrix (ECM) proteins resulting in distortion of cardiac structure and function. Early work indicated the role of ANKRD1 in non-cardiac fibrotic responses where ANKRD1 transcripts and proteins are highly induced after dermal wounding in mice and its over-expression enhanced the healing process [37]. Moreover, *Ankrd1*-knockout mice have been shown to be viable but display impaired wound healing that is characterized by dermal fibroblast dysfunction [38]. There is evidence that nuclear ANKRD1, in concert with nucleolin, functions in the wound repair process and modulates ECM remodeling via transcriptional regulation of matrix metalloproteinases gene expression [28]. Interestingly, recent work showed that aged plasminogen activator inhibitor-1 (PAI-1) knockout mice displayed significant cardiac-specific fibrosis and differential microarray gene expression profiling revealed that several genes involved in pro-fibrotic responses including *Ankrd1* were upregulated in knockout hearts compared to wild type hearts [59]. In addition, overexpression of ANKRD1 in the cytosol of Cos-1 cells has been reported to induce elevation of Early growth response gene 1 (*Egr1*), a potent zinc finger transcription factor shown to induce enhanced collagen deposition and wound repair [60]. Taken together, evidence from in vitro and cardiac specific PAI-knockout animal models suggests the involvement of the ANKRD1-Egr1 axis as a mechanistic effector of cardiac fibrosis. Interestingly, it has been reported that induced shuttling of YB-1 from a cytosolic to nuclear localization reduces renal fibrosis due to the repression of pro-fibrotic factors and activation of anti-fibrotic factors [61]. Since ANKRD1 is an interacting partner of YB-1 [18], it would be worth investigating whether the localization of ANKRD1 also affects the development of cardiac fibrosis through YB-1.

### 4.3. Cardiomyocyte Apoptosis

**Figure 3:**
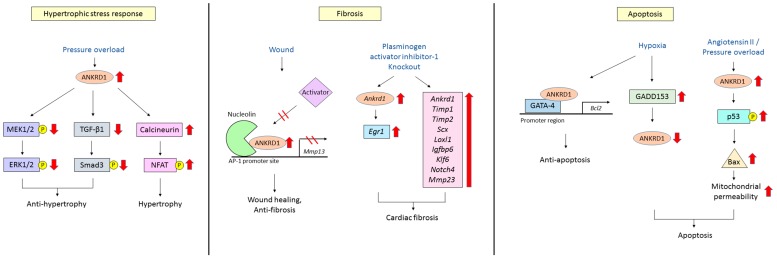
Schematic diagram showing the pathological cardiac functions of ANKRD1 in relation to hypertrophy, fibrosis and apoptosis. Hypertrophic stress response: Pressure overload rapidly increases the expression of cardiac ANKRD1 and inhibits the ERK and TGF-β/Smad pathways, leading to the attenuation of hypertrophy. This is thought to be an adaptive and protective response. Conversely, increase in ANKRD1 also exacerbates pathological cardiac remodeling through activation of the calcineurin/NFAT pathway, a signaling pathway known to have an important role in cardiac hypertrophy. Hence, the role of ANKRD1 in cardiac hypertrophy is still controversial; Fibrosis: Nuclear ANKRD1 interacts with nucleolin and hinders the binding of positive transcriptional activators to the AP-1 promoter site, thus inhibiting transcription of the *Mmp13* gene. This limits the expression of MMP13 to within appropriate physiological levels to enable successful tissue repair. On the other hand, expression of *Ankrd1* is increased in plasminogen activator inhibitor-1 knockout mouse. This in turn induces the elevation of *Egr1* which is associated with cardiac fibrosis. In addition, the concomitant upregulation of several genes involved in pro-fibrotic responses including *Ankrd1* also leads to the development of cardiac fibrosis; Apoptosis: Hypoxia-induced up-regulation of GADD153 leads to a decrease in ANKRD1 expression which has been shown to cause apoptotic cell death. ANKRD1 also acts as a co-factor of the transcription factor GATA-4 to induce anti-apoptotic *Bcl2* gene expression. These results support the role of ANKRD1 as an anti-apoptotic factor. Conversely, it has also been reported in hearts stimulated with either angiotensin II or challenged by pressure overload that overexpression of ANKRD1 led to an increase in cardiomyocyte apoptosis, indicating that ANKRD1 could also have pro-apoptotic properties. Hence, ANKRD1 seems to be able to either enhance or inhibit apoptosis under different conditions, depending on the type of pathological stimuli. Black arrows indicate the downstream effect. Red arrows indicate an increase or decrease in expression or phosphorylation of the protein. Inhibition of downstream effect is indicated by two red lines drawn across a black arrow. Yellow-circled P indicates phosphorylation. ANKRD1, ankyrin repeat domain 1; AP-1, activator protein 1; *Bcl2*, B-cell lymphoma 2; *Egr1*, early growth response gene 1; ERK, extracellular signal–regulated kinase; GADD153, growth arrest and DNA damage 153; GATA-4, GATA binding protein 4; Igfbp6, insulin-like growth factor binding protein 6; Klf6, kruppel like factor 6; Loxl1, lysyl oxidase like 1; MEK, mitogen-activated protein kinase kinase; Mmp13, matrix metalloproteinase 13; NFAT, nuclear factor of activated T-cells; Scx, scleraxis basic helix-loop-helix transcription factor; Smad, mothers against decapentaplegic homolog; TGF-β, transforming growth factor β; Timp2, tissue inhibitor of metalloproteinase 2.

Limited ischemic injury to the heart results in apoptosis of cardiomyocytes [62]. Hypoxia-induced up-regulation of growth arrest and DNA damage 153 (GADD153) leads to a concomitant decrease in ANKRD1 expression which has been shown to cause apoptotic cell death of H9c2 rat myoblast cells, suggesting that ANKRD1 is anti-apoptotic [63]. Accordingly, ectopic expression of ANKRD1 in these cells decreased hypoxia-induced apoptosis. Similar observations were observed in an in vivo rat coronary ischemia/reperfusion injury model [62]. In addition, a new study showed that ANKRD1 acts as a co-factor of the transcription factor GATA binding protein 4 (GATA-4) to induce anti-apoptosis *Bcl2* gene expression in in vitro hypoxia/reoxygenation and in vivo ischemic/reperfusion experimental models, lending further support to the role of ANKRD1 as an anti-apoptotic factor [30]. Conversely, in a recent report by Shen and colleagues, overexpression of ANKRD1 led to an increase in cardiomyocyte apoptosis when hearts were stimulated with either angiotensin II or challenged by pressure overload, indicating that ANKRD1 could also have pro-apoptotic properties [64]. It is worth noting that ANKRD1 has also been linked to an increase in apoptotic cell death in human hepatoma cells [65]. The results thus far suggest that ANKRD1 could play an important role in the regulation of apoptosis in cardiomyocytes. Intriguingly, ANKRD1 seems to be able to either enhance or inhibit apoptosis under different conditions, depending on the type of pathological stimuli. This aspect of ANKDR1 warrants further investigation. The link between ANKRD1 functions and apoptosis is further reinforced by the presence of multiple predicted caspase cleavage sites described in the following section. A summary of the pathological cardiac functions of ANKRD1 is illustrated in Figure 3.

### 4.4. ANKRD1: Disease-Causing or Cardioprotective?

Whether overexpression of ANKRD1 is a causative factor in cardiac pathology or if it is simply an adaptive response to cardiac insults is still a matter of debate. It is intriguing that on the one hand, increased expression of ANKRD1 has been found to exacerbate cardiac pathology [57], induce cardiac fibrosis [59] and increase cardiac apoptosis [64]. However, on the other hand, ANKRD1 has also been shown to protect against cardiac hypertrophy [56], cardiac fibrosis [28] and display anti-apoptotic properties [30]. These seemingly contradictory effects of ANKRD1 may be a reflection of its different actions depending on its nuclear/cytosolic subcellular localization or could be due to differences in the study design, pathological stimuli and in vitro vs. in vivo experimental platforms used in the various studies. Given the limited data available, we speculate that increased ANKRD1 expression is more of an adaptive response of the heart to pathological insults but when left unregulated, could contribute to malignant cardiac phenotype. It is hoped that future studies on ANKRD1 would be able to answer these questions.

## 5. Regulation of ANKRD1

ANKRD1 is mainly expressed in heart muscle [2]. However, its expression profile greatly differs during the early stages of heart development compared to the adult heart [18]. In human fetuses between 10 and 14 weeks old, ANKRD1 is strongly expressed in a uniform manner across all cardiac compartments [3]. As cardiogenesis proceeds, ANKRD1 expression starts to decline in ventricular tissue but remains highly expressed in the atrium [2,66]. Asymmetry of ANKRD1 expression has also been reported in newborn and early postnatal piglets where levels of ANKRD1 were found to be higher in the left myocardium as compared to the right [67]. These observations indicate that mechanisms exist to control and regulate the protein levels of ANKRD1 throughout development and into adulthood.

The expression of ANKRD1 can also be stimulated by several factors. The inducibility of ANKRD1 expression was first documented by Chu and colleagues where they observed that the cytokines interleukin-1α and TNF-α greatly increased *ANKRD1* mRNA in human vascular endothelial cells [9]. A similar observation was seen in murine myoblasts where TNF-α up-regulated *Ankrd1* mRNA and protein levels [27]. ANKRD1 levels were increased in response to a variety of stimuli including α1-adrenergic signaling, β-adrenergic stimulation and transforming growth factor β (TGF-β) signaling [68,69,70]. Furthermore, ANKRD1 expression in skeletal muscle is also induced during fatiguing exercise, skeletal muscle denervation, wound healing and angiogenesis [37,71,72].

Down-regulation of ANKRD1 expression has been documented in specific settings. For instance, *Ankrd1* mRNA was reported to be highly repressed by the cardiotoxic antineoplastic drug, doxorubicin [2]. In addition, hypoxia and heat stress decrease ANKRD1 expression [63,73]. The molecular mechanisms regulating intra-cellular ANKRD1 have not been fully elucidated. We discuss some putative mechanisms in the following sections.

### 5.1. Regulation of ANKRD1 Promoter Activity

The expression of ANKRD1 is tightly regulated by several nuclear factors. In particular, the activity of the *Ankrd1* promoter depends on an M-CAT element (5′-CATDYY-3′), an element crucially involved in Ras-dependent activation of expression of several cardiac genes [68,74]. M-CAT elements are the binding sites of the transcription enhancer factor-1 (TEF-1), a binding factor required for basal activity of the CARP promoter in cardiac myocytes and possibly also responsible for the regulation of ANKRD1 expression during stress response downstream of mitogen-activated protein kinase pathways [6,69].

The *Ankrd1* gene is also a downstream target of NK2 homeobox 5 (Nkx2.5), a cardiac-specific transcription factor [18]. Nkx2.5 directly controls the promoter activity of *Ankrd1* by binding to the 2.5 kb upstream regulating region to activate transcription [75]. In addition, Nkx2.5 also interacts with GATA-4, a zinc finger transcription factor that binds to a GATA-4 binding site in the promoter region of the *Ankrd1* gene, to mediate transcription in a cooperative manner [5].

Tumor suppressor p53 and myogenic differentiation (MyoD) transcription factors have been reported to up-regulate *Ankrd1* promoter activity in C2C12 skeletal muscle cells [29]. In addition, Smad proteins have been found to regulate *Ankrd1* transcription in smooth muscle cells [70]. Upon activation by TGF-β, Smad proteins bind to the CAGA motif located at 108 bp upstream of the transcription start site of *Ankrd1* to initiate transcription. In another study, down-regulation of *Ankrd1* transcription was reported in rat H9c2 cells where GADD153 was observed to decrease *Ankrd1* expression during hypoxia-induced apoptosis [63]. Collectively, these findings indicate that regulation of ANKRD1 expression is controlled by multiple factors which may vary between different cell types.

### 5.2. Post-Transcriptional Gene Silencing of ANKRD1

The discovery of the association of microRNAs with various cardiovascular diseases has fostered strong impetus for developing microRNA-based diagnostic markers and therapeutics [76]. MicroRNAs are non-coding RNAs that play a pivotal role in guiding the RNA-induced silencing complex (RISC) to the 3 prime untranslated region (3′UTR) of its target genes and subsequently suppressing the translational machinery or facilitating the degradation of targeted transcripts. In eukaryotic cells, microRNA genes are transcribed by RNA polymerase II as long primary transcripts and subsequent editing by nuclear ribonuclease and endonuclease within the nucleus and cytoplasm, respectively. The 22-nucleotide mature microRNAs interact with Argonaute within the RISC and exert their inhibitory effect after binding to target transcripts [77]. Mounting evidence has demonstrated microRNAs as key post-transcriptional regulators involved in almost all biological pathways in animals. In the past few years, various studies have shown that in addition to interfering with signaling cascades, microRNAs also regulate the expression of proteins that are critical for cyto-architecture assembly, as well as maintaining the integrity of the extracellular matrix, and hence contribute to the development of cardiac hypertrophy and fibrosis [78,79,80]. As discussed in previous sections, ANKRD1 was shown to play important roles in various pathological pathways that lead to cardiac hypertrophy and fibrosis, as well as apoptosis. Interestingly, several of these ANKRD1-associated signaling pathways were also reported to be modulated by microRNAs, which raise the possibility that microRNAs may modulate or cross talk with ANKRD1 and subsequently affect the signaling cascades. Using three miRNA target prediction algorithms TargetScan v7.1 (http://www.targetscan.org/) [81], miRDB v5.0 (http://mirdb.org/) [82] and miRanda (http://www.microrna.org) [83], the authors of this review found numerous microRNAs predicted to target the *ANKRD1*-3′UTR, suggesting possible involvement of microRNAs in post-transcriptional regulation of *ANKRD1* (see Table S1). Further data mining of the literature for heart failure-related microRNAs identified from various heart failure cohorts revealed eight heart failure-related microRNAs, namely miR-101, miR-129-5p, miR-139-5p, miR-17, miR-199a-5p, miR-221-5p, miR-34a-5p, and miR-545-5p, that have at least one predicted target site in *ANKRD1*-3′UTR (Table 2) [84]. Among these microRNAs, miR-199a is particularly interesting as it was found to be upregulated in myocardial biopsies from heart failure patients [85] and is involved in various pathological pathways [86,87,88]. Ironically, as discussed in the previous section, ANKRD1 was found to be upregulated in the hypertrophic heart. Hence, upregulation of miR-199a in the diseased heart may be part of a compensatory response to counter the hyper induction of ANKRD1. The other heart failure-related microRNA, miR-34a-5p, which was found upregulated in the serum of heart failure patients, is predicted to target *ANKRD1* by both TargetScan and miRanda. miR-34a-5p was shown to be an important microRNA involved in cardiac fibrosis, cardiac regeneration and apoptosis pathways through targeting various vital genes, including *Smad4*, *Bcl2*, *Cyclin D1*, and *Sirt1* [89,90]. In the future, it would be interesting to decipher the interaction between these eight heart failure-related microRNAs and *ANKRD1* in cardiac lineages and in vivo platforms.

In addition to computational microRNA target prediction, one recent study on a transcriptome-wide search for microRNA targets in human myocardial tissue was performed to uncover binding interactions between microRNAs and target genes [96]. In this study, 10 microRNA seed families were identified to bind to *ANKRD1* mRNA transcripts at various seed regions (Table 3), providing additional evidence that expression of ANKRD1 is potentially regulated by microRNAs. Noteworthy, miR-199a, was also identified by prediction algorithms and published works as previously discussed. Interestingly, three of the microRNAs, miR-1, miR-29 and miR-133, were reported to be dysregulated in cardiac hypertrophy model and heart failure [85,97], suggesting possible regulatory effects of microRNA on the expression of ANKRD1 in the diseased hearts. Although there is a current lack of experimental confirmations to support the role of microRNAs in regulating ANKRD1 expression, it would be an interesting area for further exploration and validation given that these microRNAs harbor therapeutic potential as biological targets [98].

### 5.3. Proteolytic Processing

#### 5.3.1. Calpain 3

Calpains are a family of calcium-dependent cysteine proteases that alter protein structure and function via their proteolytic processing activities. Mechanisms modulating calpain activity are known to regulate cardiac function in normal and diseased states [99]. ANKRD1 has been reported to be cleaved by calpain 3 at the N-terminal region between aa 65 and 71, a region within its coiled-coil domain which could potentially disrupt one of its NLS sequences [17]. Thus, it was suggested that cleavage of ANKRD1 by calpain 3 may possibly affect the nuclear import of ANKRD1 and hence affect its downstream gene expression regulatory functions. Furthermore, in the same study by Laure et al., cleavage of ANKRD1 by calpain 3 was also found to increase the binding efficiency between the long C-terminal portion of ANKRD1 and sarcomeric titin, again indicating that the nuclear translocation of ANKRD1 may be affected [17].

#### 5.3.2. Caspase 3

Caspases are a family of endoproteases that play important roles in programmed cell death and inflammation [100]. In mammals, caspase 3 activates the caspase signaling cascade responsible for apoptosis execution. In a proteome-wide search for caspase 3 substrates, ANKRD1 was identified to be cleaved by caspase 3 at a region near its N-terminus between residues 26–97 [101]. However, in a later study by Samaras et al., it was shown that the caspase 3 inhibitor carbobenzoxy-valyl-alanyl-aspartyl-[O-methyl]-fluoromethylketone (ZVAD-fmk) did not affect ANKRD1 degradation in adult rat ventricular myocytes [102]. Thus, the in vivo relevance of ANKRD1 cleavage by caspase 3 remains to be determined. Interestingly, prediction of the cleavage of ANKRD1 by various enzymes using the software PeptideCutter (http://web.expasy.org/peptide_cutter/) did not yield any predicted caspase 3 cleavage sites [103]. Instead, a caspase 1 cleavage site was predicted at Asp277. On the other hand, thirteen caspase cleavage sites with a prediction score >50% were found using CaspDB (http://caspdb.sanfordburnham.org) (Table 4), one at residue 277 that coincides with the caspase 1 site predicted by PeptideCutter and one at residue 30 that could be the possible caspase 3 site experimentally identified in the proteome-wide caspase substrate study mentioned earlier [101]. The presence of a caspase 1 site provides some inkling of a role of ANKRD1 in inflammatory responses. However, most of these processing sites have not been experimentally validated and remain to be verified.

### 5.4. Degradation

#### 26S Proteasome

The ubiquitin-proteasomal degradation pathway is a major mechanism used by cells to regulate the concentration of a host of proteins, providing a form of protein quality control critical for the maintenance of heart function in health and disease [105]. Appropriate regulation of protein turnover is especially important in cardiomyocytes since these cells have very limited regenerative capacity. In an attempt to investigate if the 26S proteasomal pathway affects the turnover of ANKRD1, Badi et al., treated HeLa cells (stably transfected with *ANKRD1*) or SK-MES-1 cells (human lung carcinoma) with the protein synthesis inhibitor cycloheximide (CHX) in the absence or presence of MG132, a 26S proteasome inhibitor [106]. They found that MG132 almost completely prevented CHX-mediated decay of ANKRD1, indicating that degradation of ANKRD1 is largely dependent on the 26S proteosomal pathway. The PEST motifs in ANKRD1 were also found to be responsible, at least in part, for mediating the degradation of ANKRD1 by the 26S proteasome. Moreover, ANKRD1 protein levels were not affected by incubation of the CHX-treated cells with NH_4_Cl nor the calpain pathway inhibitor calpeptin, confirming that degradation of ANKRD1 does not involve the lysosomal or calpain degradation pathways. The role of the 26S proteasome in regulating ANKRD1 protein turnover was also investigated in another two cell types—human microvascular endothelial cells (HMVEC) and adult rat ventricular myocytes (ARVM)—and similar results were obtained, further confirming the earlier findings [102]. In addition, it was previously found that ANKRD1 interacts with the muscle specific really interesting new gene (RING) finger proteins muscle RING-finger protein 1 (MuRF1) and MuRF2, which are sarcomere-associated E3 ubiquitin-ligases that catalyze the transfer of ubiquitin to protein substrates, signaling them towards proteasomal degradation [107]. Whether proteasome-mediated degradation of ANKRD1 is directly affected by MuRFs, however, has not been conclusively shown and should be further investigated. Another interesting point to note is that the half-life of ANKRD1 was observed to be significantly longer in cardiomyocytes (h) compared to non-muscle cell types (min), indicating that ANKRD1 degradation is regulated differently in different cell types, probably related to its distinct functions in muscle vs. non-muscle cells [102,106].

## 6. ANKRD1 and Cardiovascular Diseases

### 6.1. Cardiomyopathy

The up-regulation of ANKRD1 has been linked to various cardiac diseases including cardiac hypertrophy, dilated cardiomyopathy, ischemic cardiomyopathy and arrhythmogenic right ventricular cardiomyopathy [6,8,108,109]. A number of mutations in the *ANKRD1* gene have been associated with cardiac disease. In a study by Arimura et al., three *ANKRD1* missense mutations (Pro52Ala, Thr123Met and Ile280Val) were found in three patients with hypertrophic myopathy [31]. These gain-of-function mutations resulted in increased ANKRD1 binding to titin and myopalladin, leading to greatly reduced nuclear localization of ANKRD1 in the cells and thus affecting its gene regulatory functions. In another study, three missense heterozygous mutations in *ANKRD1* (Pro105Ser, Val107Leu and Met184Ile) were identified in four patients with dilated cardiomyopathy [8]. The Met184Ile mutation resulted in loss of binding of ANKRD1 to talin-1 and FHL2 while the Pro105Ser mutation led to a loss of talin-1 binding. In this case, localization of ANKRD1 in the cells was not altered but the loss of binding to talin-1 and FHL2 (both of which have important stretch-sensory functions) possibly led to a disruption of mechanical stretch-induced signaling and subsequent gene expression. A third study by Duboscq-Bidot and colleagues identified five missense mutations (Glu57Gln, Arg66Gln, Thr116Met, Leu199Arg or Ala276Val), corresponding to 2% of dilated cardiomyopathy cases studied [110]. Most of the mutations directly affected the functions of ANKRD1 by reducing its ability to repress transcription through the *MLC2v* promoter and also resulted in poorly controlled hypertrophic response towards phenylephrine.

### 6.2. Heart Failure

In patients with end-stage heart failure due to dilated, ischemic or arrhythmogenic right ventricular cardiomyopathy (ARVC), ANKRD1 levels were found to be significantly augmented in the myocardium, suggesting that up-regulation of ANKRD1 may play an important role in the disease progression of heart failure [7,109,111]. Consistent with this suggestion, induced overexpression of ANKRD1 in rats contributes to the pathogenesis of heart failure [68]. Since apoptosis is considered to play an essential role in heart failure progression, one school of thought is that the pro-apoptotic actions of ANKRD1 may be an adverse contributing factor [64]. ANKRD1 expression has also been shown to be positively correlated with pro-atrial natriuretic peptide (proANP) and brain natriuretic peptide (BNP) levels in failing animal and human hearts, suggesting that ANKRD1 expression may be mediated via common regulatory pathways [66,67,109]. Both proANP and BNP are natriuretic peptides well accepted for their diagnostic value as markers for heart failure, thus pointing to the potential of ANKRD1 as a useful reflector of the onset and progression of heart failure.

## 7. Potential as a Cardiac Biomarker

ANKRD1 has been proposed to be a potential biomarker for cardiac remodeling and disease progression in dilated cardiomyopathy, cardiac hypertrophy and heart failure [6,109,111]. We highlight some salient properties of ANKRD1 that support its potential as a cardiac biomarker. Firstly, it is predominantly expressed in cardiac myocytes with only a low basal level detected in skeletal muscle. A comparison of the mRNA expression and protein distribution profile obtained from the GeneCards^®^ database v4.4.2 (http://www.genecards.org/) (Figure S1) revealed that ANKRD1 shares very similar expression patterns with the current gold standard cardiac biomarkers—natriuretic peptides A and B and troponins-T and -I [112,113]. Secondly, ANKRD1 is rapidly induced and maintained in cardiac tissues in response to cardiac insults and is also highly expressed in the above-mentioned cardiomyopathies as well as heart failure. Thirdly, expression of ANKRD1 has also been shown to be positively correlated with that of the natriuretic peptides in the failing heart. This allows it to be potentially used as a diagnostic tool and also as a marker of disease progression. Crucially, such a marker role for ANKRD1 will depend on whether it is released into the circulation to produce plasma concentrations which have a close and dynamic relationship to the degree of cardiac injury and dysfunction. The presence of ANKRD1 in circulating blood has not been studied. We postulate that it could be released into the bloodstream as a result of cardiac injury, much in the same way as sarcomeric troponins (the current gold standard clinical biomarker for myocardial infarction) are released during myocardial injury [114]. Furthermore, there is currently a dearth of good biomarkers for cardiac fibrosis as presently known candidates have primarily inconclusive association with the disease for reasons such as lack of organ specificity. Hence, cardiac-specific scaffold proteins with nuclear actions such as ANKRD1 may represent a new group of potential new biomarkers that warrants further investigation [115]. The potential of ANKRD1 as a biomarker is certainly an exciting area for future research.

## 8. Conclusions

This review summarizes the present understanding of ANKRD1 in terms of its structure, expression profile, functions, regulation and clinical significance. Taken together, ANKRD1 is a protein with pleiotropic functions. In the nucleus, it functions as a transcriptional regulator of a wide variety of genes, while, in the cytosol, it binds to proteins of the sarcomere to maintain its structural integrity and also functions as part of a mechano-sensing unit to relay stretch signals to gene expression. Being highly induced during periods of stress and various forms of cardiomyopathies, ANKRD1 has the potential to serve as a cardiac biomarker with possible diagnostic and prognostic utility. Moving forward, a greater understanding of the mechanisms underlying its functions could possibly open up new therapeutic possibilities in combating cardiovascular diseases.

## Figures and Tables

**Table 1:** Known interacting partners of ANKRD1.

Interacting Partner	Functional Effects	Verified by	Association with Disease	Ref.
**Transcription factors**				
YB-1	negative transcriptional co-factor of YB-1; cardiomyogenesis	Y2H, Co-IP GST-pulldown	-	[18]
NF-κB	negative transcriptional co-factor of NF-κB; anti-inflammation	Co-IP	-	[27]
Nucleolin	co-repression of *MMP13* gene transcription; wound healing	Y2H, Co-IP	-	[28]
p53	positive transcriptional co-activator of p53; regulation during development and stress response	Co-IP, GST-pulldown	-	[29]
GATA-4	positive transcriptional co-activator of GATA-4; anti-apoptosis	Co-IP	-	[30]
**Structural components**				
Myopalladin	maintaining sarcomere structural integrity	Y2H, GST-pulldown	Pro52Ala, Thr123Met and Ile280Val mutations in ANKRD1 increase its binding to myopalladin and are associated with hypertrophic cardiomyopathy	[24,31]
Titin	Mechano-sensing, regulation of gene expression	Y2H, Blot overlay, Fluorescence spectroscopy	Pro52Ala, Thr123Met and Ile280Val mutations in ANKRD1 increase its binding to titin and are associated with hypertrophic cardiomyopathy	[1,31]
Desmin	Unknown	Y2H	-	[13]
Talin-1	Mechano-sensing, regulation of gene expression	Y2H	Met184Ile and Pro105Ser mutations in ANKRD1 decrease its binding to talin-1 and are associated with dilated cardiomyopathy	[8]
**Signaling molecules**				
FHL2	Mechano-sensing, regulation of gene expression	Y2H	Met184Ile mutation in ANKRD1 decreases its binding to FHL2 and is associated with dilated cardiomyopathy	[8]
CASQ2	Sequestration of CASQ2, resulting in lower Ca^2+^ concentration to regulate various signaling pathways	FLAG-pulldown, Blot overlay, Co-IP	-	[32]
14-3-3 proteins	Cytoplasmic retention of ANKRD1 and thus inhibiting its nuclear functions	GST-pulldown	-	[21]
PKCα	Sequestration of PKCα at intercalated discs	GST-pulldown, Co-IP	Sequestration of PKCα at the intercalated discs results in chronic PKCα stress signaling and is associated with heart failure	[33]

Ala, Alanine; ANKRD1, Ankyrin repeat domain 1; CASQ2, Calsequestrin 2; Co-IP, Co-immunoprecipitation; FHL2, Four-and-a-half LIM domains 2; GATA-4, GATA binding protein 4; GST, Glutathione S-transferase; Ile, Isoleucine; Met, Methionine; NF-κB, Nuclear factor κ-light-chain-enhancer of activated B cells; *MMP13*, matrix metalloproteinase 13; PKCα, Protein kinase C α; Pro, Proline; Ser, Serine; Thr, Threonine; Val, Valine; Y2H, Yeast two-hybrid; YB-1, Y-box binding protein 1.

**Table 2:** HF-related microRNAs and the number of predicted target sites on *ANKRD1*-3′UTR.

MicroRNA	Relative Expression in HF vs. Control	Sample Type	Ref.	TargetScan		miRanda	
				Conserved	Poorly Conserved	Good mirSVR Score and Conserved	Non-Good mirSVR Score and Conserved
miR-101	Up	Serum	[91]	0	0	0	1
miR-129-5p	Up	Plasma	[92]	0	1	0	1
miR-139-5p	Down	PBMC	[93]	0	0	0	1
miR-17	Up	Serum	[91]	0	0	0	1
miR-199a-5p	Up	Cardiac biopsy	[85]	0	0	0	1
miR-211-5p	Down	Whole blood	[94]	0	1	0	0
miR-34a-5p	Up	Serum	[95]	0	1	1	2
miR-545-5p	Up	Whole blood	[94]	0	1	0	0

HF, heart failure; miR, microRNA; PMBC, peripheral blood mononuclear cell; UTR, untranslated region.

**Table 3:** MicroRNA seed families identified to bind to *ANKRD1* mRNA transcript [96].

MicroRNA Seed Family	Seed Abundance	Seed Sequence
miR-1ab/206/613	0.083384693	GGAATGT
miR-133abc	0.031080626	TTGGTCC
miR-29abcd	0.023655364	AGCACCA
miR-199ab-5p	0.003286278	CCAGTGT
miR-28-5p/708/1407/1653/3139	0.003077991	AGGAGCT
miR-34ac/34bc-5p/449abc/449c-5p	0.001174053	GGCAGTG
miR-574-5p	0.000356476	GAGTGTG
miR-193a-5p	0.00024827	GGGTCTT
miR-9/9ab	0.000246405	CTTTGGT
miR-132/212/212-3p	0.000215371	AACAGTC

miR, microRNA.

**Table 4:** Caspase cleavage sites in ANKRD1 predicted from CaspDB database [104].

Residue	Amino Acid Sequence P5–P5′	Prediction Score
304	KAIFD-SLREN	0.913
150	PDVCD-EYKRT	0.875
30	EDFRD-GEYEA	0.853
258	TPLHD-AVRLN	0.788
120	TEPVD-VPTFL	0.782
183	IEFRD-MLEST	0.697
244	ACEAD-LNAKD	0.682
200	GGNLD-VLKLL	0.664
290	KTPMD-LVLHW	0.642
216	ISARD-KLLST	0.624
253	DREGD-TPLHD	0.573
277	MYGAD-LNIKN	0.561
142	KFLSD-KNNPD	0.544

## Supplementary Materials

Supplementary materials can be found at www.mdpi.com/1422-0067/18/7/1362/s1.
