# Supplementary material for: Ankyrin Repeat Domain 1 Protein: A Functionally Pleiotropic Protein with Cardiac Biomarker Potential
Source: Int J Mol Sci. 2017 Jun 26;18(7):1362. doi: 10.3390/ijms18071362 (PMC5535855; doi:10.3390/ijms18071362)
Supplement: Supplementary file 1 [file ijms-18-01362-s001.zip › ijms-199082-SI.pdf]

Table S1. MicroRNAs predicted to target ANKRD1.

**TargetScan**		**miRDB**	**miRanda**	
Conserved	Poorly Conserved	Gene (Target score)	Good mirSVR score and conserved	Non-good mirSVR score and conserved
hsa-miR-582-5p	hsa-miR-3920	hsa-miR-4729	hsa-miR-544	hsa-miR-544
	hsa-miR-4699-3p	hsa-miR-4698	hsa-miR-34a	hsa-miR-539
	hsa-miR-4698	hsa-miR-4768-3p	hsa-miR-10b	hsa-miR-191
	hsa-miR-8063	hsa-miR-4446-3p	hsa-miR-10a	hsa-miR-34a
	hsa-miR-5581-5p	hsa-miR-760	hsa-miR-425	hsa-miR-203
	hsa-miR-4297	hsa-miR-4427	hsa-miR-218	hsa-miR-10b
	hsa-miR-532-5p	hsa-miR-3160-5p	hsa-miR-300	hsa-miR-10a
	hsa-miR-6872-3p	hsa-miR-6515-3p	hsa-miR-381	hsa-miR-425
	hsa-miR-6508-3p	hsa-miR-3163	hsa-miR-33a	hsa-miR-218
	hsa-miR-4726-5p	hsa-miR-338-5p	hsa-miR-33b	hsa-miR-139-5p
	hsa-miR-4640-5p	hsa-miR-425-5p	hsa-miR-410	hsa-miR-326
	hsa-miR-6724-5p	hsa-miR-5003-5p	hsa-miR-340	hsa-miR-300
	hsa-miR-6773-5p	hsa-miR-8088	hsa-miR-873	hsa-miR-381
	hsa-miR-6774-5p	hsa-miR-6837-3p	hsa-miR-449a	hsa-miR-33a
	hsa-miR-635	hsa-miR-4509	hsa-miR-449b	hsa-miR-33b
	hsa-miR-4753-3p	hsa-miR-544a	hsa-miR-34c-5p	hsa-miR-410
	hsa-miR-6809-3p	hsa-miR-8055		hsa-miR-219-5p
	hsa-miR-942-5p	hsa-miR-493-5p		hsa-miR-340
	hsa-miR-6515-3p	hsa-miR-3074-3p		hsa-miR-873
	hsa-miR-1236-3p	hsa-miR-20a-3p		hsa-miR-181c
	hsa-miR-6818-3p	hsa-miR-6835-5p		hsa-miR-181a
	hsa-miR-6895-3p	hsa-miR-506-5p		hsa-miR-181d
	hsa-miR-593-3p	hsa-miR-146a-3p		hsa-miR-101
	hsa-miR-6069	hsa-miR-3920		hsa-miR-144
	hsa-miR-135b-5p	hsa-miR-6849-3p		hsa-miR-449a
	hsa-miR-135a-5p	hsa-miR-642b-3p		hsa-miR-449b
	hsa-miR-889-5p	hsa-miR-642a-3p		hsa-miR-34c-5p
	hsa-miR-8074	hsa-miR-4666a-3p		hsa-miR-217
	hsa-miR-28-3p			hsa-miR-376c
	hsa-miR-708-3p			hsa-miR-27a
	hsa-miR-7844-5p			hsa-miR-27b
	hsa-miR-3121-3p			hsa-miR-135a
	hsa-miR-3161			hsa-miR-135b
	hsa-miR-599			hsa-miR-371-5p
	hsa-miR-138-1-3p			hsa-miR-129-5p
	hsa-miR-138-1-3p			hsa-miR-106a
	hsa-miR-5003-5p			hsa-miR-106b
	hsa-miR-513b-5p			hsa-miR-17
	hsa-miR-6837-3p			hsa-miR-20a
	hsa-miR-412-3p			hsa-miR-20b
	hsa-miR-6754-3p			hsa-miR-93

TargetScan		miRDB	miRanda	
Conserved	Poorly Conserved	Gene<br>(Target score)	Good mirSVR score and conserved	Non-good mirSVR score<br>and conserved
	hsa-miR-6509-5p			hsa-miR-519d
	hsa-miR-331-5p			hsa-miR-339-5p
	hsa-miR-5582-3p			hsa-miR-204
	hsa-miR-548o-3p			hsa-miR-211
	hsa-miR-1323			hsa-miR-199b-5p
	hsa-miR-616-5p			hsa-miR-199a-5p
	hsa-miR-371b-5p			hsa-miR-433
	hsa-miR-373-5p			hsa-miR-149
	hsa-miR-371a-5p			hsa-miR-205
	hsa-miR-372-5p			hsa-miR-197
	hsa-miR-514b-5p			hsa-miR-488
	hsa-miR-513c-5p			hsa-miR-361-5p
	hsa-miR-3160-5p			hsa-miR-543 12
	hsa-miR-218-5p			hsa-miR-365 22
	hsa-miR-636			
	hsa-miR-6737-3p			
	hsa-miR-7157-3p			
	hsa-miR-5008-3p			
	hsa-miR-1914-5p			
	hsa-miR-10a-5p			
	hsa-miR-10b-5p			
	hsa-miR-6732-3p			
	hsa-miR-652-5p			
	hsa-miR-651-3p			
	hsa-miR-4509			
	hsa-miR-5692a			
	hsa-miR-548c-3p			
	hsa-miR-5692a			
	hsa-miR-5692a			
	hsa-miR-545-5p			
	hsa-miR-548au-3p			
	hsa-miR-34c-5p			
	hsa-miR-449b-5p			
	hsa-miR-449a			
	hsa-miR-34a-5p			
	hsa-miR-222-5p			
	hsa-miR-936			
	hsa-miR-199b-3p			
	hsa-miR-199a-3p			
	hsa-miR-3129-5p			
	hsa-miR-5094			
	hsa-miR-3074-3p			
	hsa-miR-4427			

TargetScan		miRDB	miRanda	
Conserved	Poorly Conserved	Gene (Target score)	Good mirSVR score and conserved	Non-good mirSVR score and conserved
	hsa-miR-4680-3p			
	hsa-miR-5187-3p			
	hsa-miR-493-3p			
	hsa-miR-8060			
	hsa-miR-7154-5p			
	hsa-miR-1255a			
	hsa-miR-1255b-5p			
	hsa-miR-6744-5p			
	hsa-miR-3134			
	hsa-miR-4733-3p			
	hsa-miR-1226-3p			
	hsa-miR-6511b-3p			
	hsa-miR-6511a-3p			
	hsa-miR-1178-3p			
	hsa-miR-767-3p			
	hsa-miR-6828-3p			
	hsa-miR-6728-3p			
	hsa-miR-3194-3p			
	hsa-miR-6796-3p			
	hsa-miR-3928-5p			
	hsa-miR-6806-3p			
	hsa-miR-127-5p			
	hsa-miR-3646			
	hsa-miR-381-3p			
	hsa-let-7b-3p			
	hsa-miR-300			
	hsa-let-7a-3p			
	hsa-let-7f-1-3p			
	hsa-miR-98-3p			
	hsa-miR-4666a-3p			
	hsa-miR-190a-3p			
	hsa-miR-4766-5p			
	hsa-miR-146a-3p			
	hsa-miR-760			
	hsa-miR-4747-5p			
	hsa-miR-5196-5p			
	hsa-miR-6748-5p			
	hsa-miR-6748-5p			
	hsa-miR-6756-5p			
	hsa-miR-6766-5p			
	hsa-miR-6745			
	hsa-miR-363-5p			
	hsa-miR-4483			

TargetScan		miRDB	miRanda	
Conserved	Poorly Conserved	Gene<br>(Target score)	Good mirSVR score and conserved	Non-good mirSVR score<br>and conserved
	hsa-miR-1293			
	hsa-miR-3115			
	hsa-miR-4511			
	hsa-miR-548b-3p			
	hsa-miR-3653-3p			
	hsa-miR-3658			
	hsa-miR-299-5p			
	hsa-miR-29b-1-5p			
	hsa-miR-6765-3p			
	hsa-miR-3622b-3p			
	hsa-miR-3622a-3p			
	hsa-miR-5096			
	hsa-miR-130b-5p			
	hsa-miR-6805-3p			
	hsa-miR-5691			
	hsa-miR-7158-3p			
	hsa-miR-203b-3p			
	hsa-miR-3622a-5p			
	hsa-miR-5582-5p			
	hsa-miR-4423-3p			
	hsa-miR-6806-5p			
	hsa-miR-562			
	hsa-miR-4789-3p			
	hsa-miR-642a-3p			
	hsa-miR-642b-3p			
	hsa-miR-425-5p			
	hsa-miR-5708			
	hsa-miR-4666a-5p			
	hsa-miR-600			
	hsa-miR-548j-3p			
	hsa-miR-548ae-3p			
	hsa-miR-548ah-3p			
	hsa-miR-548aq-3p			
	hsa-miR-548am-3p			
	hsa-miR-548aj-3p			
	hsa-miR-548x-3p			
	hsa-miR-548z			
	hsa-miR-548ac			
	hsa-miR-548bb-3p			
	hsa-miR-548d-3p			
	hsa-miR-548h-3p			
	hsa-miR-302a-5p			
	hsa-miR-3183			

TargetScan		miRDB	miRanda	
Conserved	Poorly Conserved	Gene (Target score)	Good mirSVR score and conserved	Non-good mirSVR score and conserved
	hsa-miR-4723-3p			
	hsa-miR-6769b-3p			
	hsa-miR-6892-3p			
	hsa-miR-4687-5p			
	hsa-miR-5685			
	hsa-miR-4690-3p			
	hsa-miR-34a-3p			
	hsa-miR-3689e			
	hsa-miR-3689b-5p			
	hsa-miR-3689a-5p			
	hsa-miR-3689f			
	hsa-miR-4679			
	hsa-miR-7974			
	hsa-miR-4675			
	hsa-miR-4741			
	hsa-miR-4446-3p			
	hsa-miR-4721			
	hsa-miR-583			
	hsa-miR-4768-3p			
	hsa-miR-4433a-3p			
	hsa-miR-4459			
	hsa-miR-506-5p			
	hsa-miR-548c-3p			
	hsa-miR-20a-3p			
	hsa-miR-544a			
	hsa-miR-6715b-3p			
	hsa-miR-1298-5p			
	hsa-miR-3977			
	hsa-miR-623			
	hsa-miR-4780			
	hsa-miR-6780b-3p			
	hsa-miR-5006-3p			
	hsa-miR-4755-5p			
	hsa-miR-204-5p			
	hsa-miR-211-5p			
	hsa-miR-6734-3p			
	hsa-miR-4297			
	hsa-miR-5581-5p			
	hsa-miR-532-5p			
	hsa-miR-3162-3p			
	hsa-miR-6748-3p			
	hsa-miR-5699-3p			
	hsa-miR-4421			

TargetScan		miRDB	miRanda	
Conserved	Poorly Conserved	Gene (Target score)	Good mirSVR score and conserved	Non-good mirSVR score and conserved
	hsa-miR-7152-5p			
	hsa-miR-3074-5p			
	hsa-miR-3124-3p			
	hsa-miR-892c-3p			
	hsa-miR-892c-3p			
	hsa-miR-3667-3p			
	hsa-miR-6734-3p			
	hsa-miR-6868-3p			
	hsa-miR-3124-3p			
	hsa-miR-34b-3p			
	hsa-miR-27a-3p			
	hsa-miR-513a-5p			
	hsa-miR-27b-3p			
	hsa-miR-5096			
	hsa-miR-6760-3p			
	hsa-miR-1208			
	hsa-miR-181b-2-3p			
	hsa-miR-181b-3p			
	hsa-miR-4420			
	hsa-miR-653-3p			
	hsa-miR-6837-3p			
	hsa-miR-6815-3p			
	hsa-miR-5580-5p			
	hsa-miR-6881-5p			
	hsa-miR-6835-5p			
	hsa-miR-6803-5p			
	hsa-miR-6751-5p			
	hsa-miR-6842-5p			
	hsa-miR-6752-5p			
	hsa-miR-7110-5p			
	hsa-miR-637			
	hsa-miR-6852-5p			
	hsa-miR-661			
	hsa-miR-6849-3p			
	hsa-miR-6512-3p			
	hsa-miR-6720-5p			
	hsa-miR-5586-5p			
	hsa-miR-4668-3p			
	hsa-miR-548c-3p			
	hsa-miR-8088			
	hsa-miR-3177-5p			
	hsa-miR-3664-5p			
	hsa-miR-4714-5p			

TargetScan		miRDB	miRanda	
Conserved	Poorly Conserved	Gene (Target score)	Good mirSVR score and conserved	Non-good mirSVR score and conserved
	hsa-miR-514a-5p			
	hsa-miR-1225-5p			
	hsa-miR-1229-5p			
	hsa-miR-3614-5p			
	hsa-miR-6500-3p			
	hsa-miR-6837-3p			
	hsa-miR-6754-3p			
	hsa-miR-412-3p			
	hsa-miR-6515-3p			
	hsa-miR-1236-3p			
	hsa-miR-6809-3p			
	hsa-miR-4753-3p			
	hsa-miR-2117			
	hsa-miR-4757-3p			
	hsa-miR-4325			
	hsa-miR-7703			
	hsa-miR-557			
	hsa-miR-507			
	hsa-miR-29a-5p			
	hsa-miR-181a-2-3p			
	hsa-miR-6509-3p			
	hsa-miR-1273g-3p			
	hsa-miR-4436b-5p			
	hsa-miR-3667-3p			
	hsa-miR-6734-3p			
	hsa-miR-4448			
	hsa-miR-6743-3p			
	hsa-miR-4433b-5p			
	hsa-miR-4433a-5p			
	hsa-miR-6834-3p			
	hsa-miR-4666b			
	hsa-miR-20a-3p			
	hsa-miR-544a			
	hsa-miR-8055			
	hsa-miR-129-5p			
	hsa-miR-452-3p			
	hsa-miR-33b-5p			
	hsa-miR-33a-5p			
	hsa-miR-3680-3p			
	hsa-miR-101-3p.2			
	hsa-let-7b-3p			
	hsa-let-7f-1-3p			
	hsa-miR-98-3p			

TargetScan		miRDB	miRanda	
Conserved	Poorly Conserved	Gene (Target score)	Good mirSVR score and conserved	Non-good mirSVR score and conserved
	hsa-let-7a-3p			
	hsa-miR-1185-2-3p			
	hsa-let-7f-2-3p			
	hsa-miR-1185-1-3p			
	hsa-miR-4789-5p			
	hsa-miR-4729			
	hsa-miR-338-5p			
	hsa-miR-4666a-3p			
	hsa-miR-338-5p			
	hsa-miR-4666a-3p			
	hsa-miR-493-5p			
	hsa-miR-3662			
	hsa-miR-3163			
	hsa-miR-4795-3p			
	hsa-miR-1303			

(A)

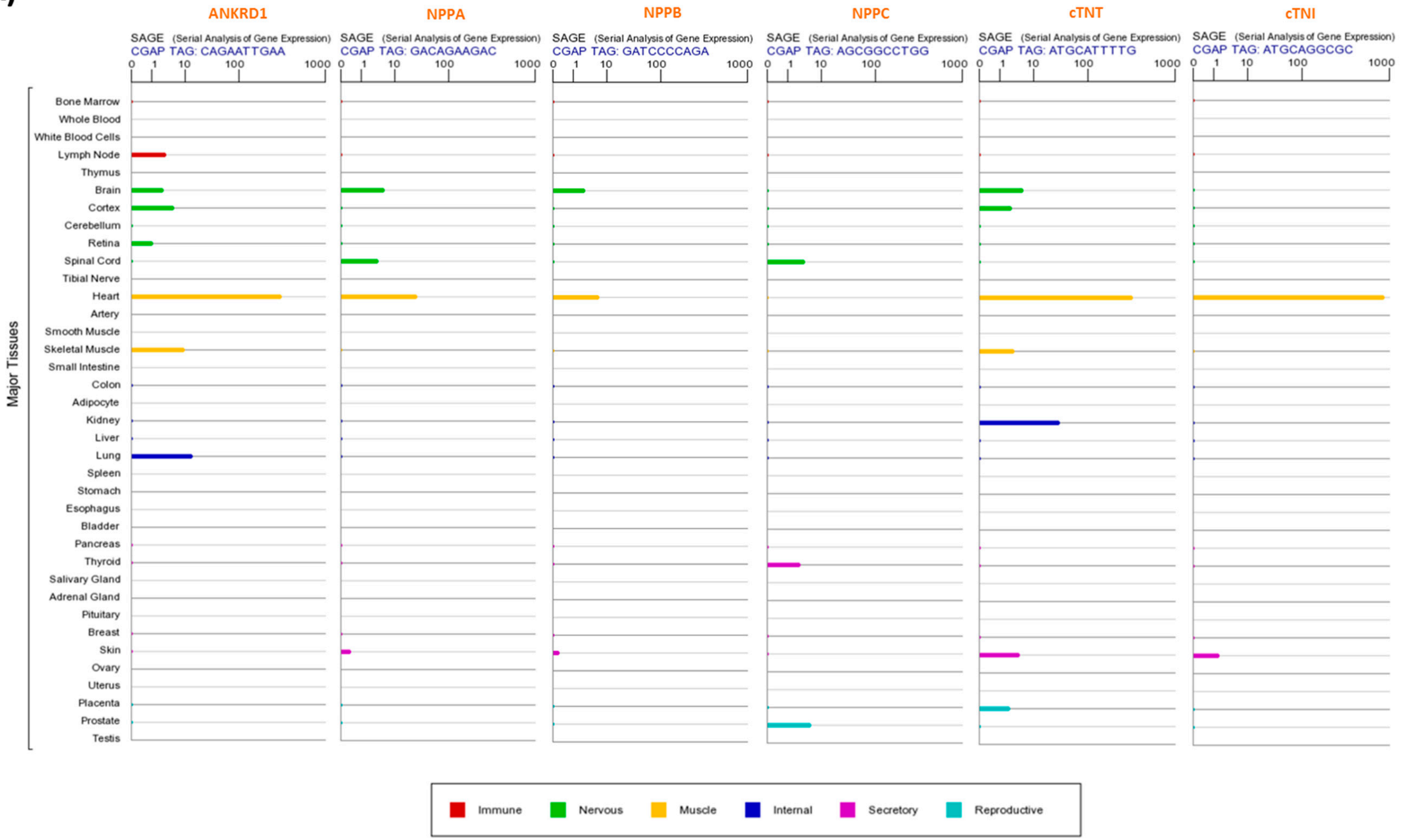
ANKRD1
NPPA
NPPB
NPPC
cTNT
cTNI
SAGE (Serial Analysis of Gene Expression)
CGAP TAG: CAGAATTGAA
CGAP TAG: GACAGAAGAC
CGAP TAG: GATCCCCAGA
CGAP TAG: AGCGGCCTGG
CGAP TAG: ATGCATTTTG
CGAP TAG: ATGCAGGCGC
0 1 10 100 1000
Major Tissues
Bone Marrow
Whole Blood
White Blood Cells
Lymph Node
Thymus
Brain
Cortex
Cerebellum
Retina
Spinal Cord
Tibial Nerve
Heart
Artery
Smooth Muscle
Skeletal Muscle
Small Intestine
Colon
Adipocyte
Kidney
Liver
Lung
Spleen
Stomach
Esophagus
Bladder
Pancreas
Thyroid
Salivary Gland
Adrenal Gland
Pituitary
Breast
Skin
Ovary
Uterus
Placenta
Prostate
Testis
Immune
Nervous
Muscle
Internal
Secretory
Reproductive

(B)

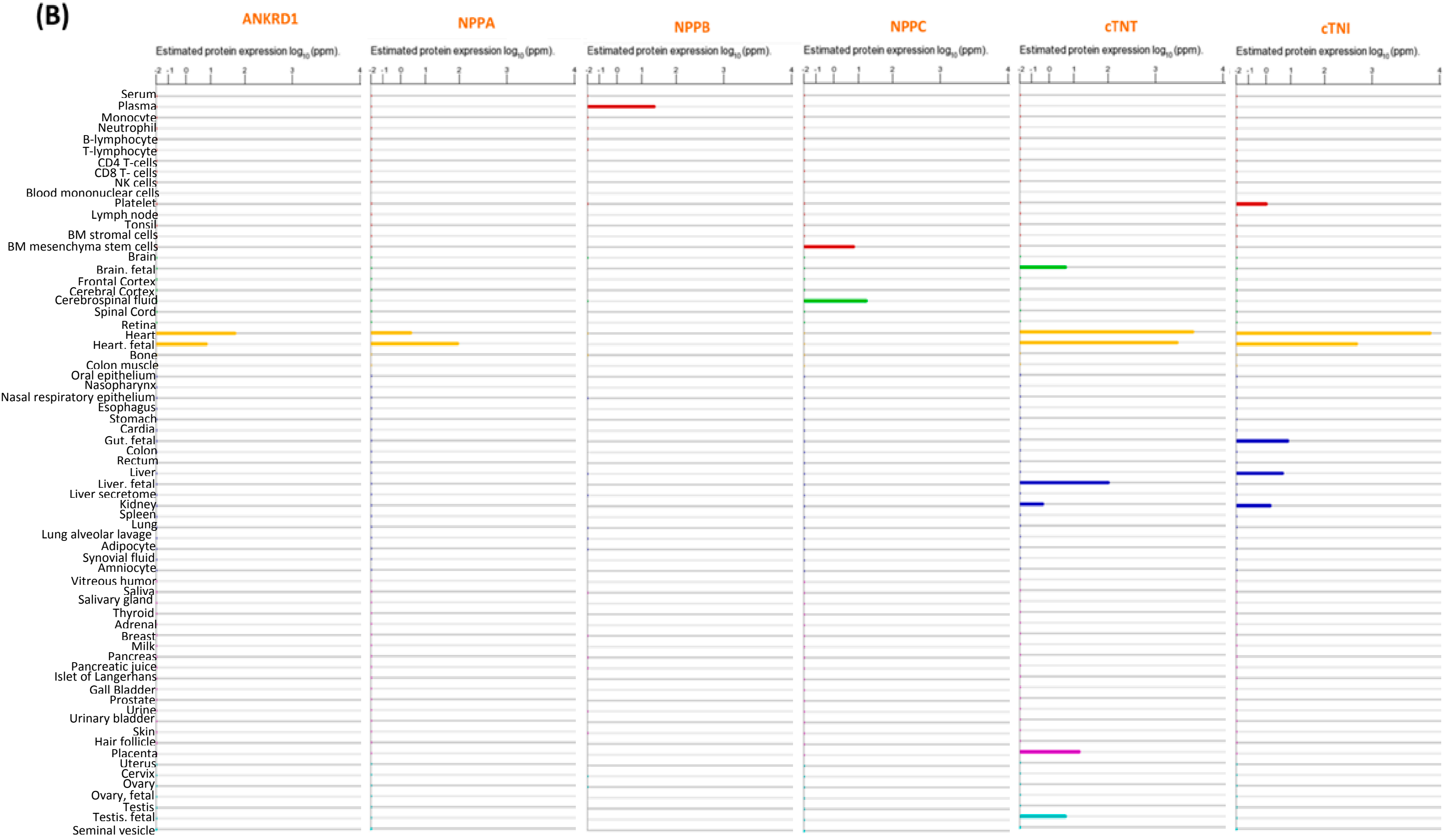

Figure S1. mRNA expression and protein distribution profiles of ANKRD1 in comparison with other established and upcoming cardiac biomarkers. (**A**) mRNA expression of various cardiac biomarkers in 19 different tissue and compartments by Serial Analysis of Gene Expression (SAGE); (**B**) Protein distribution profiles of various cardiac biomarkers in 92 tissues and compartments. Data and figures were extracted and adapted from the GeneCards® database (www.genecards.org)

[112]. ANKRD1, ankyrin repeat domain 1; BM, bone marrow; cTNI, cardiac troponin I; cTNT, cardiac troponin T; NPPA, natriuretic peptide A; NPPB, natriuretic peptide B; NPPC, natriuretic peptide C.
